# Draft genomes of *Neisseria perflava* UMB0578, *Proteus mirabilis* UMB8339, and *Enterococcus faecalis* UMB7967 isolated from urine samples

**DOI:** 10.1128/mra.00498-24

**Published:** 2024-08-20

**Authors:** Alex Kula, Ali Khan, Megan Martinez, Jevan Terry, Helen Appleberry, Alan J. Wolfe, Catherine Putonti

**Affiliations:** 1 Department of Biology, Loyola University, Chicago, Illinois, USA; 2 Bioinformatics Program, Loyola University, Chicago, Illinois, USA; 3 Department of Chemistry and Biochemistry, Loyola University, Chicago, Illinois, USA; 4 Department of Microbiology and Immunology, Loyola University Chicago, Maywood, Illinois, USA; University of Maryland School of Medicine, Baltimore, Maryland, USA

**Keywords:** *Neisseria perflava*, *Proteus mirabilis*, *Enterococcus faecalis*, urinary microbiome, urobiome, SUI, rUTI

## Abstract

The urinary tract of females harbors a variety of microorganisms, both for those with and without symptoms. Here, we present the draft genome sequences of three isolates from urine samples*—Neisseria perflava* UMB0578, *Proteus mirabilis* UMB8339, and *Enterococcus faecalis* UMB7967.

## ANNOUNCEMENT

Recent efforts have been focused on cataloging the bacterial species of the urinary microbiota (1), and research has found that many species within the female urinary microbiota also inhabit the vaginal microbiota (2–4). As the most recent catalog of bacterial species diversity in the urinary tract shows, numerous taxa can be found in this ecological niche (1). While some are considered “commensal” or “transient,” those taxa associated with symptoms/diseases are significantly better studied. In our group’s continued effort to characterize and sequence strains from the urinary microbiota, here, we present the draft genome assemblies of three species: *Neisseria perflava*, *Proteus mirabilis*, and *Enterococcus faecalis*. While *N. perflava* is not associated with urinary tract infections, both *P. mirabilis* and *E. faecalis* are known causes of both uncomplicated and complicated urinary tract infections (UTIs) (5).

*N. perflava* UMB0578 was isolated from a catheterized urine sample from a female with stress urinary incontinence [IRB: LU204133, LU 204658, and LU206449 (Loyola University Chicago) (6)]. Both *P. mirabilis* UMB8339 and *E. faecalis* UMB7967 were isolated from voided urine samples from two different females diagnosed with recurrent UTI [IRB: 170077AW (University of California, San Diego) (7, 8)]. These strains were isolated using the expanded quantitative urine culture method (9); the taxonomy of these isolates was determined via matrix-assisted laser desorption ionization-time of flight, as previously described (10), and stored at −80°C in the Loyola Urinary Education and Research Collaborative (LUEREC) collection. We obtained samples from this collection and streaked the *N. perflava* isolate onto a nutrient broth (NB) agar plate and the *P. mirabilis* and *E. faecalis* isolates onto brain heart infusion agar plates. The *N. perflava* NB plate was incubated at 37°C for 48 hours; the other two plates were incubated at 35°C in 5% CO_2_ for 24 hours. For each, a single colony was selected and grown in their respective culture media under the above culture conditions. DNA extraction was performed using the DNeasy Blood and Tissue Kit (Qiagen), following the protocol for Gram-positive organisms, and sent to SeqCoast Genomics (Portsmouth, NH) for library construction, using the Illumina DNA Prep tagmentation kit, and sequencing on the Illumina NextSeq2000 platform. Raw reads (2 × 150 bp reads) were assembled using BV-BRC v3.35.5 (11) with the “auto” parameter. The raw reads were first trimmed using Trim Galore v0.6.5 (https://github.com/FelixKrueger/TrimGalore) and then assembled using Unicycler v0.4.8 (12). Assemblies were next polished with Pilon v1.23 (13). BV-BRC also was used to calculate genome coverage, completeness, and contamination. Genome annotations were produced using the NCBI Prokaryotic Genome Annotation Pipeline v6.7 (14). Unless otherwise specified, default parameters were used for all software tools.

Genome assembly statistics are provided in Table 1. Continued efforts to sequence both commensal and pathogenic species of the urinary microbiota are needed to better understand this community, both for symptomatic and asymptomatic individuals.

**TABLE 1:** Genome assembly statistics

Strain	UMB0578	UMB8339	UMB7967
Species	*N. perflava*	*P. mirabilis*	*E. faecalis*
SRA accession no.	SRR28710915	SRR28710898	SRR28710894
Assembly accession no.	JBCGFA000000000	JBCGEX000000000	JBCGEU000000000
No. raw reads	1,699,568	1,535,364	3,035,810
Assembly length (bp)	2,273,827	3,722,531	2,908,311
G+C (%)	48.98	38.49	37.3
No. contigs	42	62	43
Contigs N50 (bp)	232,257	217,641	603,203
Coverage (×)	106.01	51.49	140.04
Completeness (%)	100	100	100
Contamination (%)	0.2	0	0

## Data Availability

Table 1 lists the SRA accession numbers and assembly accession numbers for the three strains.
